# Condylar growth after non-surgical advancement in adult subject: a case report

**DOI:** 10.1186/1746-160X-5-15

**Published:** 2009-07-20

**Authors:** Antonino Marco Cuccia, Carola Caradonna

**Affiliations:** 1 Section of Orthodontics, Department of Dental Sciences "G. Messina", University of Palermo, Via del Vespro 129, 90127, Palermo, Italy

## Abstract

**Background:**

A defect of condylar morphology can be caused by several sources.

**Case report:**

A case of altered condylar morphology in adult male with temporomandibular disorders was reported in 30-year-old male patient. Erosion and flattening of the left mandibular condyle were observed by panoramic x-ray. The patient was treated with splint therapy that determined mandibular advancement. Eight months after the therapy, reduction in joint pain and a greater opening of the mouth was observed, although crepitation sounds during mastication were still noticeable.

**Conclusion:**

During the following months of gnatologic treatment, new bone growth in the left condyle was observed by radiograph, with further improvement of the symptoms.

## Background

The temporomandibular joint (TMJ) is a complex joint essential for speech, mastication and swallowing.

The mandibular condyle is an ovoidal bony structure that articulates with the temporal bone by means of a biconcave disk.

Both articular surfaces are covered by a connective fibrous tissue (condylar cartilage). On the articular surface of the condyle, the collagen fibres are parallel to the condylar surface, and are in continuity with the fibrous layer of the periosteum.

The condylar cartilage covers very dense undifferentiated mesenchyme, within which are multipotential cells, forming either cartilage or bone, depending upon the environmental circumstances [1]. The bony tissue forms the deepest part.

The TMJ grows and functions in an environment of mechanical forces that interact with cells and tissues. These forces (muscular activity, mastication, swallowing) influence the shape of mandibular condyle, through the process of biological adaptation termed "remodeling" [2].

Condylar resorption (CR) is a specific condition that affects TMJs. A number of local and systemic pathologies may cause mandibular CR. Local factors include osteoarthritis, reactive arthritis, avascular necrosis, infection, traumatic injuries and temporomandibular disorders (TMD). CR may also be due to systemic connective tissue or autoimmune diseases including rheumatoid arthritis, psoriatic arthritis, scleroderma, systemic lupus erythematosus, Sjögren syndrome, ankylosing spondylitis, and others [3-5].

Changes in condylar morphology have also been observed in experimental protrusion or retrusion of the jaw, in surgical induction of disc displacement and experimental disc perforation [6-9].

In this paper, we report a case of an adult male with TMD and left CR, in which, after occlusal modification, new bone growth in the left condyle was observed.

## Case presentation

A 30 year old male was referred to our department with a 4 years history of pain (pain scale VAS 80) and crepitus in the left TMJ during mastication, increased left facial pain, and limited functional mandibular movements.

Bruxism was reported by the patient for a period of about 18 months. He had natural molar contacts in each dental quadrant, and no parodontal disorders.

**Figure 1:**
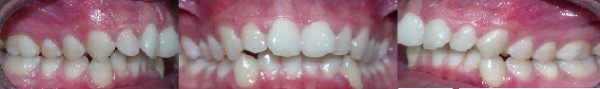
**Occlusal relationship of the patient's dentition (top: right side, middle: front view, bottom: left side)**.

Intraoral examination revealed a bilateral Class II molar relationship and a severe overjet [Figure 1]. The lower dental midline deviated to the left of the upper by 4 mm. Moderate crowding was observed in both arches.

**Figure 2:**
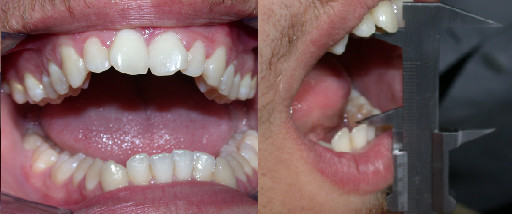
**Pre-treatment maximal active mouth opening**.

Clinical examination confirmed acute muscular pain, lateral deviation of the mandible to the left during opening and closing of the mouth, persistent pain and crepitus in left TMJ, limited opening (interincisal distance 20 mm), lateral movement to the right (3 mm), lateral movement to the left (7 mm), and difficulty protruding the mandible [Figure 2]. Crepitus and pain were determined by palpation of both joints during maximal protrusion and maximum mouth opening.

**Figure 3:**
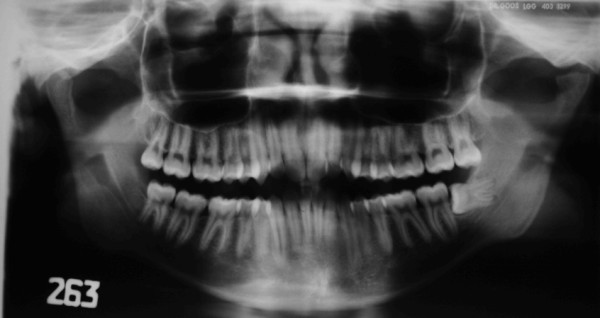
**Pre-treatment panoramic radiograph showing normal morphology of right condyle and left condylar resorption**.

A panoramic radiograph of the patient's jaws prior to removal of the mandibular left third molar, revealed left CR [Figure 3]. This type of radiographic examination does not offer as clear and reliable images as those of other techniques such as computerized or linear tomography, but does demonstrate the condyles with a degree of clarity [10].

Routine haematological analysis did not reveal any evidence of underlying systemic bone disease such as rheumatoid arthritis.

Treatment was anterior repositioning of the mandible with a hard acrylic splint in the maxilla.

**Figure 4:**
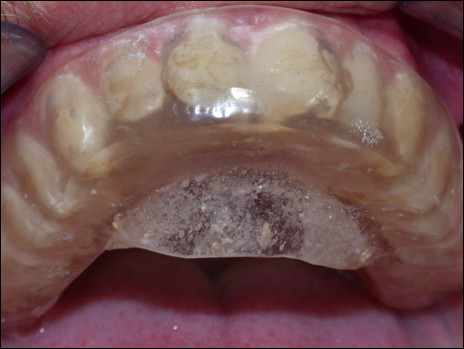
**Splint used for occlusal rehabilitation**.

The splint was 3 mm thick, and it was constructed with an inclined plane for mandibular advancement of 2,5 mm, re-centring the lower deviated dental midline [Figure 4]. The splint surface was adjusted to obtain a balanced muscular activity, and checked with conventional clinical control of the dental contacts.

**Figure 5:**
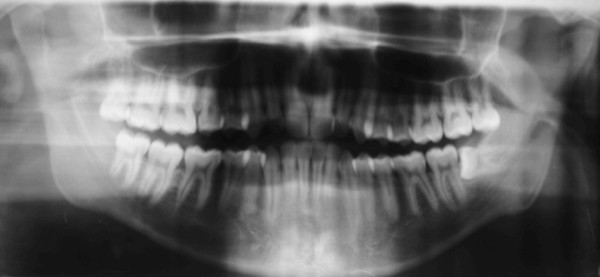
**Panoramic radiograph revealing enhanced density of the cortical layer over the left condyle 8 months after commencement of treatment**.

**Figure 6:**
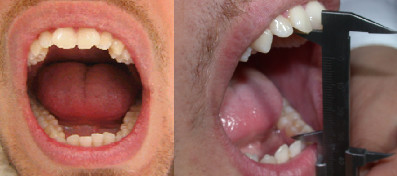
**Maximal active mouth opening after 8 months**.

**Figure 7:**
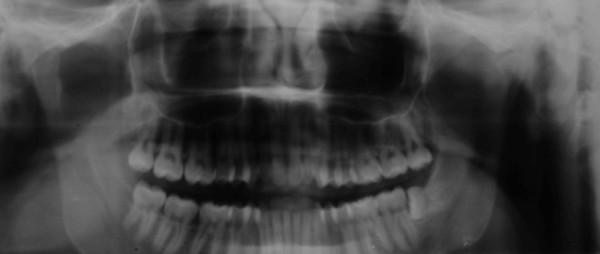
**Panoramic radiograph revealing new growth in the left condyle after 18 months of therapy**.

**Figure 8:**
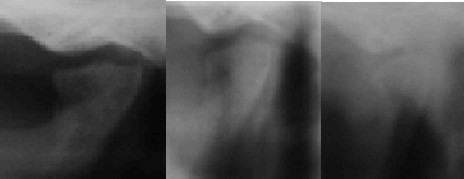
**Close-up view of the left condyle**.

The splint was used consistently, though due to work commitments, not in the mornings. The patient was reviewed every month and showed progressive symptomatic improvement on each occasion. After 8 months, a new panoramic radiograph confirmed new bone formation on the condylar surface [Figure 5]. Clinical features were improved, with reduced pain (pain scale VAS 20) and an increase in mouth opening (30 mm), although deviation of the mandible and crepitus were still evident during mastication [Figure 6]. After 18 months there was complete resolution of the symptoms, with no pain, and similar morphology of both condyles [Figure 7, 8]. At completion of treatment, there were no occlusal abnormalities.

## Discussion

Mandibular condylar cartilage is characterised histologically as fibrocartilage containing a layer of pre-chondroblastic mesenchymal stem cells which can undergo rapid differentiation into chondrocytes [11,12].

Other forms of mature articular cartilage do not have such progenitor cells and only poorly responsive chondrocytes [13].

This structural difference between mandibular condylar cartilage and hyaline articular cartilage may explain the relative difference in their regenerative potential.

The growth of mandibular condylar cartilage may be influenced by exogenic factors including mechanical factors.

These phenomena are also present in the adult, though to a lesser extent [14], since subcondylar trabecular bone formation is apparently not affected by age [15].

Animal exprerimentaion confirms that mandibular advancement causes cellular changes in rats' condyles with increased neo-vascularization and new bone formation significantly higher or equal to the levels towards the end of growth spurt [16]. Recently, McNamara *et al. *reported histological changes associated with mandibular advancement in adult Rhesus monkeys. In these monkeys, adaptive changes of the condylar cartilage were evident after 3 weeks of advancement. Furthermore, the dimensions of the condylar cartilage showed a gradual increase throughout the experimental period, whereas an untreated control group had a bony outer layer [17]. Furthermore, Rabie *et al. *found that 60-day forward mandibular positioning causes adaptive morphological changes in the condylar head of adult rats [18].

In particular, bone deposition was differential, occurring not on the anterior surface of the condyle but only on the posterior and superior surfaces, with compensatory resorption along the posterior surface of the post-glenoid tubercle, and the insertion of the lateral pterygoid muscle into the neck of the condyle [19].

Several authors have suggested that CR is possibly related to orthodontic treatment [20,21], but no previous orthodontic treatment was reported by this patient.

TMD or bruxism (that may cause TMD), may cause degenerative disease of the TMJ [22]. A rewiew of our patient's clinical data revealed that he had suffered from TMD for about 4 years and from bruxism for about 18 months.

No evidence of any bone-involving systemic diseases such as rheumatoid factors and hyperparathyroidism were found in this patient. However, it is not known how long the changes of mandibular bone structure had existed, since the condylar alteration was first noted in the patient's x-ray prior to the extraction of the left wisdom tooth. It is probable that the excessive loads produced by the force of bruxism or TMD were the causes of CR in this particular case.

Yamada *et al. *have found that the flattening of the condylar head was the most frequent unilateral condylar change. Furthermore, these authors noted that CR may be related to a lateral mandibular shift and a retrognathic mandible in patients who demonstrate TMD symptoms [23].

The capacity of TMD to remodel after acute or chronic trauma, can be used clinically not only in the correction of skeletal malocclusion, but also in treating occlusal disorders.

Splint therapy is one modality for the management of TMD. In this case, the use of a full coverage occlusal splint with mandibular advancement brought about an improvement of the clinical symptoms and new bone growth was evidenced radiographically after 18 months. The occlusal splint can correct the effects of muscle microtrauma and associated symptoms of pain or discomfort of TMJ, and also improve jaw support, as well as facilitating the spatial re-orientation of the jaw into an optimal position.

Mandibular advancement stimulates a differentiation of proliferative zone cells into chondroblasts with significant morphological changes in the TMJ [8].

Historically, treatment for CR included, apart from occlusal splint to minimize joint loading (with or without orthodontics and/or prosthetic therapy), arthroscopic lysis and lavage, condylectomy and condylar replacement with a costochondral graft, removal of hyperplastic synovial and bilaminar tissue with disk repositioning and ligament repair, and orthognathic surgery (to correct only the functional and aesthetic facial deformity) [24-27].

## Conclusion

TMJ rehabilitation of patients with CR requires careful treatment planning.

Studies suggest that increasing age and altered loading may diminish condylar growth capacity of the TMJ.

Although aging may diminish the capacity for condylar growth, this case suggests that careful mandibular repositioning can positively influence the process of remodelling of the condyle.

## Consent

Written informed consent was obtained from the patient for publication of this case report and accompanying images. A copy of the written consent is available for review by the Editor-in-Chief of this Journal.

## Competing interests

The authors declare that they have no competing interests.

## Authors' contributions

AMC and CC carried out the case study. AMC wrote the article. Both authors read and approved the final manuscript.
